# 

**Published:** 1877-03

**Authors:** 


### CONTENTS.

COMMUNICATIONS.

On Dentine . . . . . . . . . . 97
Women in Dentistry . . . . . . 100
Nutrition . . . . . . . . . . 105

MISCELLANEOUS.

Supreme Court of the United States . . 111
Limits of the Surgeon's Responsibility . . 125
The Results of Nerve Injury . . . . 130
How to Tie a Surgeon's Knot . . . . 131
Correspondence . . . . . . . 133
Dental Materia Medica and Therapeutics . . 134
Paralysis Treated by Nerve Stretching . . 136
The Anæsthetic Effects of Cold Water . . 137

EDITORIAL.

The Pennsylvania Journal of Dental Science . 138
Commencement . . . . . . . . 138
Meeting . . . . . . . . . . 139
Genuine "Old Fashioned Gold Foil." . . . 139
Iowa State Dental Society . . . . 140
Bibliographical . . . . . . . . 140

